# What makes a plant-based diet? a review of current concepts and proposal for a standardized plant-based dietary intervention checklist

**DOI:** 10.1038/s41430-021-01023-z

**Published:** 2021-10-21

**Authors:** Maximilian Andreas Storz

**Affiliations:** Center for Complementary Medicine, Department of Internal Medicine II, Faculty of Medicine, University of Freiburg, Freiburg, Germany

**Keywords:** Nutrition, Public health

## Abstract

Within the last decades, plant-based diets have received increasing interest for their potential benefits to human and environmental health. The concept of plant-based diet, however, varies widely in its definition. Current definitions range from the exclusion of all animal products to diets that include meat, fish, and dairy in varying quantities. Therefore, the main objectives of this review were twofold: (a) to investigate how researchers use the term plant-based diet in nutrition intervention studies and (b) what types of food a plant-based diet may include. Searching two databases, we found that the term “plant-based diet” evokes varying ideas to researchers and clinicians. Fifty percent of the retrieved studies that included a plant-based dietary intervention completely proscribed animal products and used the term plant-based diet interchangeably with a vegan diet. In contrast, an ~33% of trials included dairy products and 20% of dietary interventions emphasized a semi-vegetarian dietary pattern. Based on specific examples, we point out how the usage of the umbrella term “plant-based diet” may cause significant ambiguity. We often encountered incomplete descriptions of plant-based dietary interventions, which makes comparison and reproducibility of studies difficult. As a consequence, we urge others to use the term “plant-based diet” only in conjunction with a detailed dietary description. To facilitate this process, we provide a template of a standardized plant-based intervention reporting checklist. Finally, the present review also highlights the urgent need for a consensus definition of the term plant-based diet and its content.

## Introduction

Chronic diseases are increasing in global prevalence and a leading cause of mortality in the world [1, 2]. Heart disease, metabolic syndrome, and type-2-diabetes are driven by unhealthy consumption patterns, including refined sugars, processed meats, and high-fat foods [3]. More recently, however, Western societies noticed an increasing interest in plant-based eating patterns that may favorably affect chronic diseases [4, 5]. Meat’s central place in the menus is being increasingly challenged [6], whereas plant-based diets, (emphasizing grains, vegetables, fruits, legumes, nuts, and seeds), are becoming increasingly popular [7, 8].

However, according to Williams and Patel, the concept of “plant-based diet” varies widely in its definition [9]. Some definitions of “plant-based diet” exclude all animal products [10, 11] while others emphasize that plant-based eating patterns “maximiz[e] consumption of nutrient-dense plant foods while minimizing processed foods, oils, and animal foods” [12]. A popular and widely accepted definition by Ostfeld recently pointed out that a plant-based diet excludes all animal products [11], whereas other researchers emphasized that a plant-based dietary pattern may include fish, poultry, and yogurt [13, 14].

Several well-known authorities in the field of nutrition use the term “plant-based diet” synonymously with the term “vegan diet” - implying automatically that a plant-based diet is characterized by the avoidance of all flesh foods and animal-derived ingredients [15, 16]. In contrast, other sources explicitly highlight that a plant-based diet does not mean “being vegetarian” [17] or vegan [18].

**Fig. 1:**
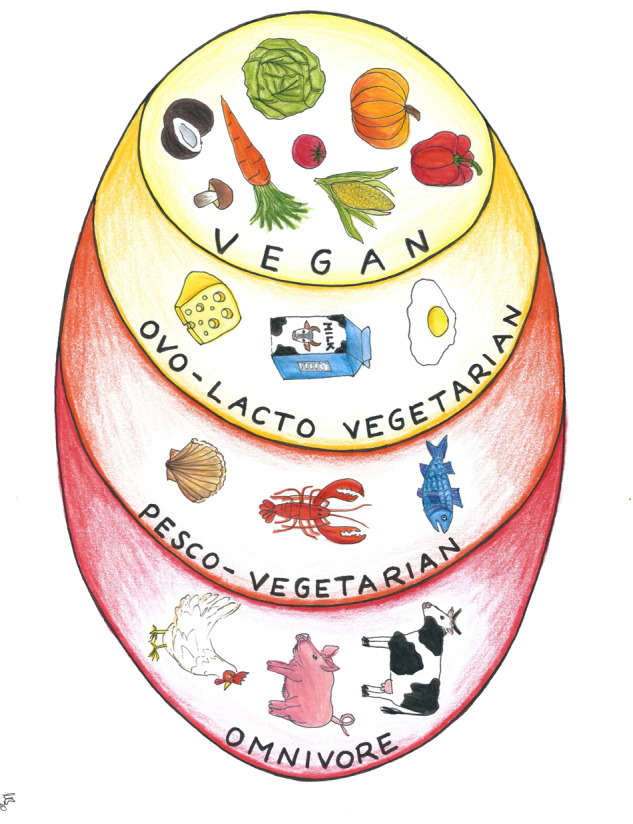
The spectrum of diets including none or only certain types of animal-based products. From right to left: vegan diet (excluding all flesh foods and animal products), lacto-ovo-vegetarian diet (excluding meat, fish, or poultry but including eggs and dairy), pesco-vegetarian diet (excluding meat or poultry but including fish) and omnivorous diet (containing all food groups) [56]. Modified from Medawar et al. [4].

The term plant-based diet is a rather new term that has been introduced by the scientific community to describe eating patterns that emphasize a large proportion of plant-dominant foods [19]. Examples include both a vegan and a vegetarian diet (Fig. 1).

The inconsistent usage of the term “plant-based diet”, however, may cause confusion and ambiguity among researchers, clinicians and the general public. Questions arise as to whether a plant-based diet should, by definition, include animal products or not? What does the term plant-based diet actually refer to and how can physicians advocate for something that is not clearly defined?

Since the term “plant-based diet” appears to evoke substantially varying concepts to researchers and clinicians, this review investigated how the medical community uses this term in scientific publications. The main objectives of this review were twofold: (a) to understand how researchers define the term “plant-based diet” in nutrition intervention studies and (b) how frequently this term is used interchangeably with other diets that are more clearly defined (e.g. the vegan diet, a lacto-ovo-vegetarian diet, a pesco-vegetarian diet, etc.).

## Methods

The electronic database of PubMed was searched using the keywords “Diet”, “Nutrition”, “Intervention” and “Plant-based” combined into the following search string: “Plant-based AND (Diet OR Intervention OR Nutrition)”. We applied the filter “Clinical Trial” and considered only English language articles. Original articles and case reports were included in this review; reviews were not considered. Reference lists of the included articles were manually screened for additional studies to ensure that all relevant trials were identified. In addition to that, we used Google Scholar’s “cited by” function to broaden our search. This allowed us to identify additional studies that could not be retrieved from controlled databases [20]. We applied no time restriction. The entire review process was conducted by the author (MAS).

Articles were included if they reported a plant-based dietary intervention in human subjects, irrespective of age, gender, race, or ethnicity. To be eligible, articles had to include the term “plant-based diet” or a synonymous term (e.g. “plant-based dietary intervention”, etc.) in the abstract, the introduction or the methods section. We considered only interventions with a minimum duration of 1 week. Studies were included irrespective of their outcome, as we focused solely on the description of each intervention. We also included studies that combined the dietary intervention with other lifestyle modifications (e.g. smoking cessation, exercising, or meditation). Moreover, studies were included regardless of their setting and location (e.g. corporate setting, in- or outpatient setting, etc.).

We did not consider animal studies for this review. Studies that investigated the effects of a single group of plant foods (e.g. plant milks) or of supplements or a particular bioactive plant compound were excluded, as well. In addition to that, surveys about plant-based diets were not considered for this review.

We identified 153 articles that met the aforementioned criteria. Eligible articles were carefully screened for a definition of the term “plant-based diet” and for a description of the dietary intervention. In a second step, we examined whether animal products were allowed as part of the plant-based diet and whether the term “plant-based diet” was used interchangeably with other terms that are more clearly defined (Fig. 1).

Finally, we assigned the prescribed diet in each study to one of five pre-defined dietary groups (including the vegan diet, the lacto-ovo-vegetarian diet, the pesco-vegetarian diet, the semi-vegetarian diet and the omnivorous diet). For example, a diet focusing on whole grains, legumes and vegetables but also containing dairy products was assigned to the lacto-ovo-vegetarian group. The intention behind this process was to gain a better understanding of the concept of “plant-based diet” and to examine how the term is used in scientific studies. Figure 1 includes descriptions of the pre-defined dietary groups. We analyzed all data using Microsoft Excel (2016).

## Results

**Fig. 2:**
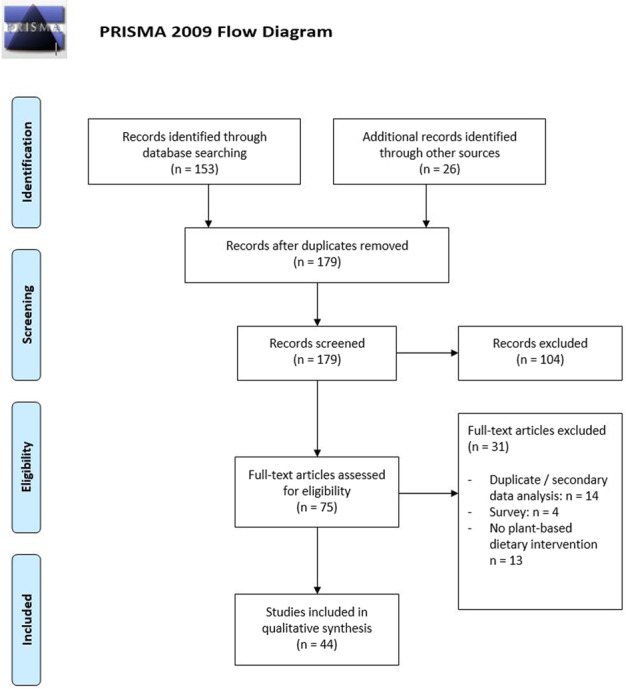
PRISMA 2009 Flow Diagram. The PRISMA flow diagram for the present review detailing the number of identified records, the number of records screened and the full texts retrieved.

The initial search using the electronic database of PubMed yielded 153 articles published between 1992 and October 2020. We identified an additional 26 records by manually screening the reference lists of the retrieved articles and by the usage of Google Scholar. The reference management software “Zotero” (Roy Rosenzweig Center for History and New Media. (2016) Zotero Computer software) was used to identify potential duplicates. We screened 179 records in total. After examination of abstract and title, 75 records remained eligible for full-text review (Fig. 2).

Occasionally, multiple studies reported different outcomes of the same intervention (e.g. when authors performed a secondary data analysis). We included only one report when multiple publications were linked to the same intervention or the same group of participants. In case of any doubts, we contacted the authors of the respective publications via email to confirm that the sample was the same.

The search revealed a total of 44 intervention studies that investigated the effects of a plant‐based diet (Table 1). We identified 37 clinical studies and 7 case reports. Table 1 provides an overview of the study characteristics (in a reversed chronological order) and shows how the term “plant-based diet” was used in the respective studies.

**Table 1:** What makes a plant-based diet? An overview of plant-based dietary interventions.

Author (year)	Location	Type	Duration	PBD defined or described	Animal foods allowed?	Meat allowed?	Fish allowed?	Dairy allowed?	Increased consumption of plant foods?	Reduced consumption of animal foods?	Intervention characteristics	Diet group	Outcome
Singh et al. (2020) [57]	USA	Single-arm trial	5 months	Yes	Yes	No	Yes	Yes	Yes	Yes	Culturally tailored PBD, based on a four tiered food guide. Highest tier included whole plant foods with minimal processing, allowing a pesco-vegetarian pattern.	Pesco-vegetarian	A PBD was deemed useful for weight management in overweight/obese Hispanic/Latino children.
Jakše et al. (2020) [58]	Slovenia	Single-arm trial	–	Yes	No	No	No	No	Yes		WFPBD. Restriction of refined fats. Ultra-processed foods omitted. No animal products. Supplemented diet.	Vegan diet	A WFPBD favorably affects weight control management and may reverse obesity.
Crimarco et al. (2020) [59]	USA	RCT	3 weeks	Yes	No	No	No	No	Yes		PBD (vegan) intervention. Participants encouraged to avoid meat, fish, poultry, eggs, and dairy products.	Vegan diet	Significant weight loss within 3 weeks; improved attitude toward PBDs.
Morin et al. (2019) [60]	Canada	Single-arm trial	12 weeks	Yes	No	No	No	No	Yes		WFPBD. Participants encouraged to avoid animal products (elimination) and to limit highly processed foods.	Vegan diet	A WFPBD favorably affects cardiovascular health (e.g. body weight, serum lipids).
Lederer et al. (2019) [21]	Germany	RCT	4 weeks	Yes	No	No	No	No	Yes		Authors use the terms strict vegan diet and PBD interchangeably. Vegan diet excludes all animal products.	Vegan diet	Vegan diet reduces cholesterol and vitamin B12 intake after 4 weeks.
Drost et al. (2019) [61]	USA	CR	3 weeks	Yes	Yes	Yes	Yes	Yes	Yes	Yes	WFPBD encouraging nutrient-dense plant foods while minimizing processed and animal-based foods.	Semi-vegetarian diet	WFPBD improves pre-operative glycemic control in persons with type-2-diabetes.
Chiba et al. (2019) [62]	Japan	Single-arm trial	–	Yes	Yes	Yes	Yes	Yes	Yes	Yes	PBD described as a “lacto-ovo-semi-vegetarian diet”, including fish once a week and meat once every 2 weeks. Eggs, milk, and plain yoghurt were used.	Semi-vegetarian diet	PBD (combined with induction therapy) favorably affects relapse rates in patients with ulcerative colitis.
Campbell and Liebman (2019) [63]	USA	CR	–	Yes	No	No	No	No	Yes		Intervention based on a food guide detailing appropriate groups of food for a WFPBD. Foods to avoid included meat, dairy, and eggs.	Vegan diet	Improved weight and hyperphosphataemia in a patient with chronic kidney disease.
Campbell et al. (2019) [64]	USA	Single-arm trial	–	Yes	No	No	No	No	Yes		WFPB nutritional approach strictly excluding animal-based foods and minimizing processed foods.	Vegan diet	WFPBD promotes weight loss and reduces blood pressure/cholesterol.
Allen et al. (2019) [65]	USA	CR	–	Yes	No	No	No	No	Yes		PBD emphasizing whole grains, vegetables, legumes; excluding all animal products and limiting packaged and processed foods	Vegan diet	WFPBD promotes weight loss and improved symptoms of heart failure and glycemic control.
Valdez et al. (2018) [26]	USA	Single-arm trial	10 days	Yes	–	–	–	–	Yes	Yes	WFPBD with minimal processed foods and saturated fat. Vegan options provided at a local restaurant. It remains unclear if animal products were allowed.	Not attributable	WFPBD improved lipid profiles in college students and altered their dietary decisions toward more plant foods.
Towery et al. (2018) [66]	USA	Single-arm trial	8 weeks	Yes	Yes	No	No	Yes	Yes	Yes	The PBD consisted of grains, fruits, vegetables, legumes, dairy products, and eggs. Processed foods and beverages discouraged.	Lacto-ovo-vegetarian diet	PBD decreased pain and improved quality of life in individuals with chronic musculoskeletal pain.
Ramal et al. (2018) [67]	USA	RCT	6 months	Yes	Yes	–	–	–	Yes	Yes	Authors describe their PBD as a high-fiber, low-fat diet, derived from mostly plant-based sources. It remains unclear which animal products were allowed.	Not attributable	PBD significantly improved glycemic control in Latinos living in medically underserved areas.
Najjar et al. (2018) [68]	USA	Single-arm trial	4 weeks	Yes	No	No	No	No	Yes		PBD excluding all animal products and emphasizing raw fruits, vegetables, avocado and raw seeds.	Vegan diet	PBD mitigated cardiovascular risk factors and reduced medication needs.
Kahleova et al. (2018) [22]	USA	RCT	16 weeks	Yes	No	No	No	No	Yes		Term “low-fat, PBD” used interchangeably with a (low-fat) vegan diet consisting of vegetables, grains, legumes, and fruits. Animal products and added oils excluded.	Vegan diet	PBD associated with reductions in body weight. fat mass, and improved insulin resistance in overweight adults.
Beauchesne et al. (2018) [69]	USA	CR	–	Yes	No	No	No	No	Yes		PBD emphasizing raw fruits, vegetables, whole grains, and legumes; excluding all animal products, added sugar, oil and salt, and highly processed foods	Vegan diet	Improved symptoms of cardiovascular disease and reduced medication burden in an 82-year-old man with cardiovascular disease.
Wright et al. (2017) [46]	New Zealand	RCT	6 months	Yes	No	No	No	No	Yes		Low-fat WFPBD that encouraged starches and avoided refined oils and animal products. Authors discouraged high-fat plant-foods and processed foods.	Vegan diet	WFPBD significantly improved BMI, cholesterol, and other risk factors in patients with metabolic disorders or heart disease.
Null and Pennesi (2017) [70]	USA	SGT	12 weeks	Yes	No	No	No	No	Yes		Anti-inflammatory PBD with 70% raw and 30% lightly cooked foods, eliminating refined carbohydrates, dairy, meat, poultry, and shellfish.	Vegan diet	A PBD along with other lifestyle and behavior modifications may provide benefits for moderate to severe depression and anxiety.
Gonciulea and Sellmeyer (2017) [71]	USA	RCT	6 weeks	Yes	Yes	No	No	Yes	Yes	Yes	Authors compared four different diets, including a soy and a non-soy PBD. Description refers to the non-soy PBD emphasizing grains, vegetables, fruits, and nuts. Eggs and dairy included.	Lacto-ovo-vegetarian diet	A soy and a non-soy plant-based diet both reduced total cholesterol and low-density lipoprotein.
Evans et al. (2017) [72]	USA	SGT	3 weeks	Yes	No	No	No	No	Yes		PBD focusing on fruits, vegetables, whole grains and legumes, seeds, and nuts. Animal products (including eggs) excluded.	Vegan diet	PBD improved total cholesterol in 74% of participants. 53% of participants lost weight.
Choi et al. (2017) [73]	USA	CR		Yes	No	No	No	No	Yes		WFPBD including vegetables, fruits, whole grains, legumes, and nuts; excluding all animal-derived foods including eggs, dairy, and meat.	Vegan diet	WFPBD improved heart failure symptoms and left ventricular ejection fraction in a 79-year-old-man.
Yadav et al. (2016) [74]	USA	RCT	12 months	Yes	No	No	No	No	Yes		Authors used a low-fat, plant-based diet based on starchy plant-foods. Meat, fish, eggs, dairy products, and vegetable oils prohibited.	Vegan diet	PBD did not significantly improve brain MRI, relapse rate, or disability in patients with multiple sclerosis.
Massera et al. (2016) [30]	USA	CR		Yes	No	No	No	No	Yes		WFPBD including vegetables, fruits, whole grains, and legumes. No animal products.	Vegan diet	WFPBD improved heart failure symptoms and left ventricular ejection fraction.
Massera et al. (2015) [27]	USA	CR		Yes	–	–	–	–	Yes		WFPB consisting of fruits, vegetables, whole grains, and legumes. Not clarified whether animal products were allowed or not.	Not attributable	WFPBD improved symptoms of angina, and cardiovascular risk factors in a 60-year-old man.
Macknin et al. (2015) [75]	USA	Prospective randomized trial	4 weeks	Yes	No	No	No	No	Yes		PBD emphasizing whole grains and plants; limiting avocado and nut intake. Subjects instructed to avoid animal products and added fat.	Vegan diet	A PBD beneficially affected BMI, blood pressure, and total cholesterol in children aged 9–18 years.
Guthrie and Bogue (2015) [76]	USA	SGT	8 weeks	Yes	Yes	Yes	Yes	Yes	Yes	Yes	Participants instructed to choose unrefined, low-glycemic whole plant foods as majority of intake. Animal products allowed as condiments.	Semi-vegetarian diet	The intervention was associated with significant weight loss but did not significantly alter serum lipids.
Clinton et al. (2015) [77]	USA	RCT	6 weeks	Yes	No	No	No	No	Yes		WFPB diet consisted of fruits, vegetables, legumes, and grains. Animal products were proscribed and the use of unrefined foods was encouraged.	Vegan diet	Significantly improved self-assessed measures of functional status among and pain in patient suffering from osteoarthritis.
Bunner et al. (2015) [78]	USA	RCT	20 weeks	Yes	No	No	No	No	Yes		Low-fat PBD omitting animal products, limiting fat intake, favoring low-glycemic index foods. The diet focused on vegetables, fruits, grains, and legumes.	Vegan diet	Improved body weight, electrochemical skin conductance, and pain in patients with type-2-diabetes and diabetic neuropathy.
Turner-McGrievy et al. (2014) [31]	USA	RCT	8 weeks	Yes	–	–	–	–	–	–	Authors carefully differentiate the umbrella term PBD and discuss the different included dietary patterns, including a vegan diet, a vegetarian diet and a pesco-vegetarian diet	Not attributable	PBD approaches for weight loss interventions does not lead to participants who are significantly different from those who enroll in standard, behavioral weight loss studies.
Bunner et al. (2014) [23]	USA	Randomized crossover study	36 weeks	Yes	No	No	No	No	Yes	Yes	“Low-fat PBD” used as a synonym for a (low-fat) vegan diet in the abstract. Crossover trial with washout period. Dietary description referring to the vegan period only (16 weeks).	Vegan diet	PBD approach may be a useful part of migraine treatment to reduce worst headache pain and average headache intensity in adults with migraine.
Mishra et al. (2013) [24]	USA	RCT	18 weeks	Yes	No	No	No	No	Yes	–	“Low-fat PBD” used as a synonym for a (low-fat) vegan diet in the abstract and introduction. Vegan diet emphasizing whole grains, vegetables, legumes, and fruits. Animal products proscribed.	Vegan diet	A PBD intervention in a corporate setting improved body weight, plasma lipids, and, in individuals with diabetes, glycemic control.
Snyder et al. (2009) [28]	USA	RCT	1 year	Yes	–	–	–	–	Yes	–	Authors used a low-saturated fat PBD. Diet characterized by an increased daily vegetable and fruit intake and a reduced saturated fat intake (goal: ≤10% of total calories from saturated fat). Not clarified whether animal products were allowed or not.	Not attributable	Authors report recruitment challenges in older cancer survivors when offered a home-based multi-behavior intervention focusing on exercise and the aforementioned diet.
Merrill and Aldana (2009) [79]	USA	RCT	4 weeks	Yes	Yes	Yes	–	Yes	Yes	Yes	PBD with little dairy intake and meat consumption. Participants encouraged to eat whole grains, legumes, vegetables, and fruits. Diet described by low fat, sugar and animal protein content.	Semi-vegetarian diet	Significant increase in daily intake of fruit, vegetables, and grains; significant decrease in dairy products and meat.
Frattaroli et al. (2008) [81]	USA	SGT	12 weeks	Yes	Yes	No	No	Yes	Yes	Yes	Very low fat, plant-based, whole foods diet, high in complex carbohydrates, low in simple carbohydrates. Including fruits, vegetables, grains, legumes, and one cup of nonfat-dairy and egg whites. From: Daubenmier et al. [80]	Lacto-ovo vegetarian diet	A combination of stress management, moderate exercise and diet improved cardiac risk factors, quality of life, and lifestyle behaviors in patients with coronary artery disease.
Frattaroli et al. (2008) [29]	USA	RCT	12 months	Yes	–	–	–	–	Yes	–	The term “(low-fat) plant-based diet” appears in the abstract and is used as a synonym for a “vegan diet” (including fruits, vegetables, whole grains), supplemented with soy, fish oil, vitamins, and selenium.	Not attributable	A combination of stress management, moderate exercise, and diet might be able to avoid or delay conventional treatment in patients with early-stage prostate-cancer.
Saxe et al. (2006) [82]	USA	Single-group intervention trial	6 months	Yes	Yes	Yes	Yes	Yes	Yes	Yes	PBD encouraging whole grains, vegetables, fruits, and legumes and to decrease meat, dairy, and refined carbohydrates. No strict avoidance of animal products.	Semi-vegetarian diet	Adoption of a plant-based diet reinforced by stress management training may have therapeutic potential and attenuate disease progression in prostate cancer patients.
Gardner et al. (2005) [83]	USA	RCT	4 weeks	Yes	Yes	No	No	Yes	Yes	Yes	Authors compared a plant-based low-fat diet to a convenience-oriented low-fat diet. The low-fat PBD emphasized vegetables, legumes, whole grains, and fruits. Diet included butter, cheese, and eggs.	Lacto-ovo-vegetarian diet	A low-fat diet including nutrient-dense plant-based foods beneficially affects total cholesterol and low density lipoprotein cholesterol.
Colombo et al. (2005) [85]	Italy	RCT	4.5 months	Yes	Yes	Yes	Yes	Yes	Yes	Yes	Authors use the term PBD for a diet emphasizing fruits in vegetables. Detailed description in Berrino et al. [84]	Semi-vegetarian diet	A plant-based diet improves serum fatty acid profile and decreases reactive oxygen metabolites.
Barnard et al. (2005) [25]	USA	RCT	14 weeks	Yes	No	No	No	No	Yes		The term “(low-fat) plant-based diet” appears in the abstract and is used as a synonym for a vegan diet consisting of vegetables, fruits, grains, and legumes. Animal products proscribed.	Vegan diet	An ad-libitum low-fat, vegan diet was associated with significant weight loss in overweight postmenopausal women.
Koebnick et al. (2004) [87]	Germany	RCT	4 weeks	Yes	Yes	No	No	Yes	Yes	Yes	PBD based on the Wholesome Nutrition recommendations (Hoffmann et al. [86]) Preference of foods of plant origin (a primarily ovo-lacto-vegetarian diet), processed as little as possible. Sparse use of fat.	Lacto-ovo vegetarian diet	The aforementioned diet improved serum lipids and exerted hypocholesterolemic effects.
Spiller et al. (2003) [88]	USA	RCT	4 weeks	Yes	Yes	Yes	Yes	–	Yes	Yes	Authors compared the effects of different almonds in the context of a PBD. Participants encouraged to replace animal foods with plant foods. Consumption of meats, poultry, and eggs limited.	Semi-vegetarian diet	Unblanched almonds may play an effective role in cholesterol-lowering, plant-based diets.
Koertge et al. (2003) [89]	USA	Single-group intervention trial	12 months	Yes	Yes	No	No	Yes	Yes	Yes	Low-fat, whole-food PBD.<10% of total calories from fat (predominantly fruits, vegetables, grains, legumes, nonfat dairy, and egg whites).	Semi-vegetarian diet	A combination of exercise, diet, social support, and stress management improved plasma lipids, blood pressure, body weight, and exercise capacity.
Saxe et al. (2001) [90]	USA	Single-group intervention trial	4 months	Yes	Yes	Yes	Yes	Yes	Yes	Yes	PBD low in saturated fat and high in fiber, emphasizing whole grains, legumes, vegetables, seeds, and fruit. Processed and refined products and foods of animal origin strictly limited.	Semi-vegetarian diet	A plant-based diet in conjunction with stress reduction decreased the rate of PSA increase in patients with recurrent prostate cancer.
Yamashita et al. (1998) [34]	Australia	Two-group intervention trial	16 weeks	Yes	Yes	Yes	Yes	Yes	Yes	Yes	PBD emphasizing soybean as the main source of protein (at least 5 days weekly). PBD was explicitly not vegetarian, but allowed chicken and fish.	Omnivorous diet	The aforementioned PBD and a meat-based diet equally lead to weight loss and metabolic benefits in overweight women.

(“What makes a plant-based diet? An overview of plant-based dietary interventions”).

*USA* United States of America, *RCT* Randomized-controlled trial, *CR* Case report, *SGT* Singe-group trial, *PBD* Plant-based diet, *WFPBD* Whole-food plant-based diet.

The majority of the included studies was done in the United States of America. Other countries of origin included (in an alphabetical order): Australia, Canada, Germany, Italy, Japan, New Zealand and Slovenia. More than 2/3 of the included studies were published after 2010 (*n* = 31/44), reflecting the aforementioned growing interest in plant-based diets within the scientific community. We refrained from calculating the mean duration of the studies because this review also includes multiple case reports (*n* = 7) without a precise duration.

All studies included either a definition or a short description of the term “plant-based diet” (*n* = 44/44). However, the descriptions of the dietary interventions varied significantly in detail. In several cases it was impossible for us to determine which foods were in- and excluded in a particular plant-based dietary intervention.

Table 1 shows bulleted summaries of the dietary interventions. Five studies used the term “plant-based diet” interchangeably with the term “vegan diet” [21–25]. Fifty percent (*n* = 22/44) of the included studies completely proscribed animal products. More than 1/3 of studies (*n* = 17/44) included animal products as part of plant-based diet. Occasionally, the dietary description was inconclusive and it was not exactly specified to which extent animal products were allowed [26–29].

For example, the authors of a 2008 study used a vegan diet that excluded all animal products but supplemented it with fish oil [29]. Thus, the (dietary) intervention was technically not vegan. Another example is a 2018 study by Valdez et al. [26]. The authors investigated the feasibility of engaging college students in a 10-day plant-based dietary intervention. The intervention emphasized the value of a whole-food plant-based diet and minimized processed foods, saturated fats and added sugars. This was also represented in the provided meals at a local restaurant offering whole foods plant-based (vegan) options. While the intervention was presumably vegan, the authors did not clarify whether animal foods were “only” minimized or fully excluded.

**Fig. 3:**
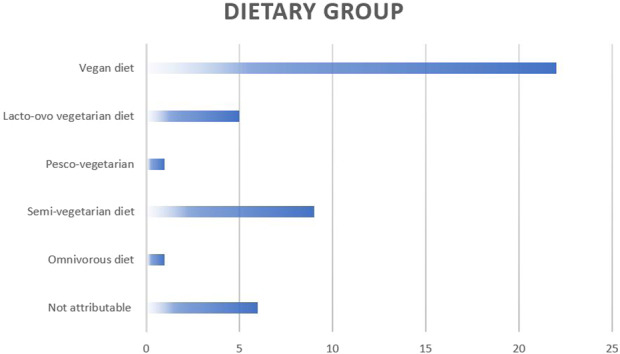
Assignment of the intervention diets to one of five pre-defined dietary groups (including a vegan diet, a lacto-ovo-vegetarian diet, a pesco-vegetarian diet or a semi-vegetarian diet). Based on missing or inconclusive data, assignment was not possible in few cases.

The literature research also revealed a case report by Massera et al., who reported a whole-food plant-based diet to reverse angina without medication or interventional procedures [27]. The dietary intervention consisted primarily of vegetables, fruits, whole grains, potatoes, beans, legumes, and nuts. Again, it was not fully clear whether small amounts of animal products were allowed or proscribed. Based on the studies’ reference list and other studies of this particular group [30], however, one may assume that the dietary intervention was technically vegan. Nevertheless, all 4 aforementioned articles were not considered in the final dietary group assignment (see below and Fig. 3).

Finally, the authors of a 2014 study carefully dissected the umbrella term “plant-based diet” and discussed the different dietary patterns used in their study. The study included a vegan diet (excluding all animal products), a vegetarian diet (excluding meat and seafood), a pesco-vegetarian diet, a semi-vegetarian diet and an omnivorous diet [31]. This study was not considered in the final dietary group assignment.

Approximately 20% (*n* = 9/44) of the retrieved studies allowed participants to consume meat and fish during the plant-based dietary intervention. Moreover, a plant-based diet contained dairy products in 34% (*n* = 15/44) of the included studies. Increased consumption of plant foods was a feature of all plant-based dietary interventions in all studies (*n* = 44/44). Almost 30% (*n* = 13/44) of studies included a “whole-food” aspect and used the prefix “whole-food” to describe the plant-based dietary intervention in greater detail.

In the last step, we assigned the prescribed diet in each study to one of five pre-defined dietary groups. These groups included a vegan diet, a lacto-ovo-vegetarian diet, a pesco-vegetarian diet, a semi-vegetarian diet and an omnivorous diet. In a few cases (*n* = 6), an attribution was impossible due to missing information or inconclusive dietary descriptions. As displayed in Fig. 3, the majority of studies prescribed a technically vegan diet. It is noteworthy that ~20% of studies allowed participants a semi-vegetarian diet, including fish and meat products.

## Discussion

The term “plant-based diet” evokes different ideas to researchers, scientists and clinicians. The primary aim of this review was to gain a better understanding of how scientists and clinicians define this term. Moreover, we sought to investigate how the term plant-based diet is used in scientific publications and nutrition intervention studies. A broad search strategy revealed 44 studies reporting a plant-based dietary intervention. Fifty percent of the included studies completely proscribed animal products. In ~20% of the retrieved studies, a plant-based diet included meat and fish. One-third of studies allowed the consumption of dairy products. While the majority of trials prescribed a technically vegan diet, 20% of trials included a semi-vegetarian eating pattern.

Our review confirmed the hypothesis that the term “plant-based diet” is used inconsistently within intervention studies. We also demonstrated that researchers have varying ideas about the content of a plant-based diet. Concepts range widely from a traditional vegan diet (excluding all animal-derived products) to a semi-vegetarian diet or even an omnivorous diet.

These findings may have important scientific and clinical implications. Clear definitions of a term or concept are necessary to allow for scientifically sound and reproducible results. According to Kampourakis, any kind of scientific discourse “has to involve concepts, the meaning of which ought to be clear among those participating in the discourse” [32]. The greater the flexibility in definitions and concepts, the less likely research findings are to be true [33]. In contrast, adherence to common standards and clear definitions is likely to reduce bias.

In the worst case, the absence of a clearly defined concept may lead to diametrically opposed results in scientific studies. This can be easily translated into clinical practice and is shown hereafter with the aid of two specific examples.

In 1998, Yamashita et al. compared two (isoenergetic) diets designed to lead to weight loss in 36 overweight or obese women in a 16-week parallel-design trial [34]. One arm of the study emphasized red meat and the other arm emphasized soybeans as the major protein source. Participants with a preference for daily meat consumption were allocated to the first arm. The second arm included subjects with a preference for plant foods who (habitually) ate more chicken and fish than red meat. Nutrients calculated from planned menus revealed a cholesterol content of 54 mg/1000 kcal in the second group. This serves an indirect indicator that their diet contained substantial amounts of animal products, because strict plant-based diets are usually much lower in cholesterol [35]. The authors found that weight loss was equal with both diets and concluded in their abstract that weight loss “occurred equally with the meat-based and plant-based diet” [34].

Seven years later, Barnard et al. published the results of a randomized clinical trial which examined the effects of a low-fat plant-based diet on body weight and metabolism [25]. Sixty-four postmenopausal, overweight women were randomly assigned to either a (low-fat) vegan diet or a control diet (based on the National Cholesterol Education Program guidelines). Adoption of a low-fat, vegan diet was associated with a mean weight loss of 5.8 (±3.2) kg in 14 weeks. Weight loss in the intervention group was significantly greater than in the control group (3.8(±2.8) kg) that regularly consumed meat and other animal products.

The two studies revealed contradicting results but were both published under the same umbrella term “plant-based diet” [25, 34]. The basic dilemma could not be clearer. In one of the studies, the term “plant-based diet” was used interchangeably with a vegan diet [25], whereas, in the other trial, the usage of the term “plant-based diet” implied the regular consumption of fish and chicken [34]. Although both diets were very different with regard to their food composition, the results were published under the same umbrella term.

The lack of a clear definition of the term ‘plant-based diet’ and its inconsistent usage may cause significant ambiguity among researchers and the public. The term ‘plant-based diet’ may therefore only be useful in the context of a clear definition and a thorough description of the applied dietary pattern. Otherwise, studies including “plant-based diets” are difficult to compare and hard to reproduce.

**Fig. 4:**
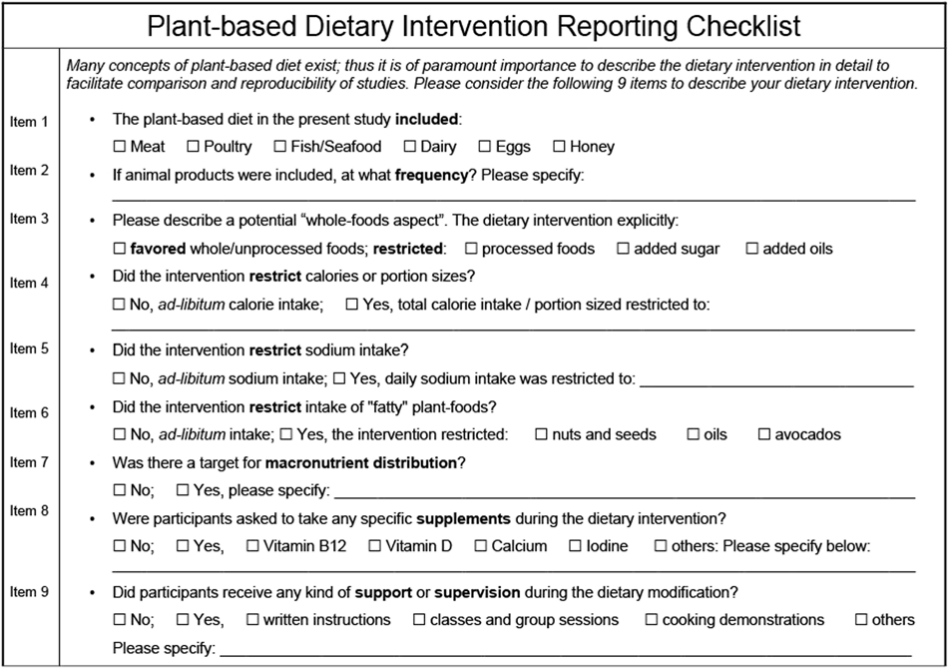
Template: the plant-based dietary intervention reporting checklist.

Reproducibility of research, however, is a fundamental tenet of good science and requires meticulous and complete reporting of interventions parameters [36]. This is particularly true for nutrition interventions, that vary from study to study in many methodological details [37]. To facilitate comparison (and reproducibility) of studies, we call for a standardized plant-based intervention reporting checklist. A template including nine items that primarily focuses on the description of the dietary intervention itself is provided below (Fig. 4).

Finally, one must pose the question whether it is justified to call a diet “plant-based” when it contains fish and chicken (at least) twice per week (as it was the case in Yamashita et al.) [34]. Should a plant-based diet contain animal products after all and if so, to what extent? What makes a plant-based diet and how much “plant-based” is necessary to exert health benefits? In the absence of a clear definition, a seemingly endless number of questions arise during a scientific discourse about plant-based dietary interventions. Although this could indeed stipulate valuable scientific discussions, one may not forget about the public health and environmental aspects behind this controversy, which have become particularly urgent during the last decades.

There is now a general consensus that diets link environmental and human health [38]. The global transition towards diets high in animal products, ultra-processed foods, and refined sugars exacts a heavy toll on planetary and human health [39, 40]. Diets high in saturated fat and meat products were frequently linked to a variety of chronic conditions, including obesity and type-2-diabetes [5, 41]. Moreover, they were associated with excessive land use, depletion of natural resources and a loss of biodiversity [40, 42]. Promoting animal-free diets that are abundant in land-sparing foods (such as vegetables) is therefore essential to boost environmental protection and human health [43, 44].

In this context, Fresán and Sabaté highlighted the alignment of environmental outcomes and human health for plant-sourced foods [39]. Plant foods are usually less resource-intensive than animal foods [45]. In addition, they were associated with beneficial effects on cardiovascular and metabolic disorders [4, 46, 47]. Plant-based diets are characterized by a reduced caloric density and a high nutrient density [48]. They also improve gut microbiota symbiosis [48], insulin sensitivity [49], beta-cell function [49, 50] and increase postprandial energy expenditure [51]. The improved postprandial metabolism after plant-based meals [52] and the reduced energy density of plant-based diets are two of the main reasons why this dietary pattern was frequently linked to weight loss [48, 49].

Reducing meat and animal product consumption is an effective way to adopt a healthier diet while simultaneously strengthening environmental protection. To promote plant-based eating patterns, however, large and well-designed public health campaigns are necessary. Physicians play an important role in this process as they are often seen as nutrition authorities and are well-positioned to deliver dietary advice and nutritional prescriptions [53, 54]. Another discussion about the value of plant-based nutrition could be a significant barrier to this development. Unfortunately, inconsistent usage of the term “plant-based diet” in the absence of clear definition of the term may exactly lead to such a discussion.

Therefore, it appears of utmost importance that future plant-based dietary interventions declare exactly to which extent animal products were included. A “plant-based dietary intervention” that includes a semi-vegetarian or even an omnivorous diet may lead to “false-negative” results (no health benefits) when compared to a plant-based diet that includes a vegan or lacto-ovo-vegetarian regimen.

Plant-based diets (and vegetarian diets in particular) are nowadays generally perceived in a positive light [55]. Dissonance about the term and its content should be resolved quickly to avoid potentially arising confusion in the general public. Otherwise, there will be a call for additional research examining the beneficial health and environmental effects of diets low (or free) in animal products. This call would inevitably translate into a substantial loss of time in the race against the growing burden of chronic non-communicable diseases and human-made environmental destruction. Thus, the authors of this paper finally argue that the term ‘plant-based diet’ should only be used in conjunction with a clear definition and a thorough description of its content.

This review has several strengths and limitations that warrant further consideration. The methodology employed in this review included a simple but easily reproducible search strategy with clearly defined in- and exclusion criteria. The outcome-independent search strategy revealed a broad spectrum of different studies. The biggest strength of this review is probably that its findings are of high translational value and highly applicable to future research studies in the field of plant-based nutrition. Our review revealed a problematic trend that requires a fast solution to allow for a better comparison between studies. The provided checklist may serve as an important guide to facilitate this process.

This review also has important limitations. Since the search strategy mainly relied on the electronic databases of PubMed and Google Scholar, it is not inconceivable that potentially relevant studies from other sources were missed. Moreover, it is likely that the English language restriction may also have limited the results.

## Conclusion

The concept of “plant-based diet” varies widely in its definition and evokes varying ideas to researchers and clinicians. Many researchers use this term interchangeably with a vegan diet, as 50% of the included studies completely excluded animal products. In contrast, a noticeable amount of trials included dairy products or emphasized a semi-vegetarian dietary pattern. We argue that this inconsistent usage of the term “plant-based diet” may cause significant confusion and makes comparison of studies difficult. Therefore, we call for a rapid consensus definition. In the meantime, we suggest to use the term “plant-based diet” only in conjunction with a detailed dietary description. Our provided checklist may support researchers and clinicians in this process.
